# Oral Health Consequences of Smokeless Tobacco Use: A Narrative Review

**DOI:** 10.7759/cureus.92625

**Published:** 2025-09-18

**Authors:** Kalina Bogdanska, Marta Kubik, Maksym Mazur, Adam Dudek, Wojciech Szkudlarek, Aleksander Bogdański, Marcin Bursy, Jan Linkiewicz, Adela Bogdanska

**Affiliations:** 1 Medical Education, Wroclaw Medical University, Wroclaw, POL; 2 Medicine, Poznan University of Medical Sciences, Poznan, POL; 3 Medicine, University Hospital in Poznań, Poznań, POL; 4 Medicine, Medical Center HCP, Poznań, POL; 5 Department of Ophthalmology, Charité – Universitätsmedizin Berlin, Berlin, DEU; 6 General Practice, University Hospital in Poznan, Poznań, POL; 7 Medical School, Poznan University of Medical Sciences, Poznań, POL; 8 Medical Education, Wroclaw Medical University, Wrocław, POL

**Keywords:** gingival recession, leukoplakia, nicotine pouches, oral mucosal lesions, oral squamous cell carcinoma, smokeless tobacco, snus

## Abstract

Smokeless tobacco (SLT), including snus, chewing tobacco, naswar, and nicotine pouches, is widely used and often perceived as a safer alternative to smoking. Among current users of SLT, mucosal alterations-most commonly smokeless-tobacco keratosis and white plaque-like lesions-are frequently observed at product placement sites, such as between the lip and gum, with patterns closely linked to the frequency and duration of use. Several reports describe partial or complete regression of these lesions after discontinuation. Periodontal findings are mixed: increased gingival recession - particularly at anterior sites corresponding to product placement - appears consistently, whereas links with deeper periodontal destruction are inconsistent and often confounded by oral hygiene and concomitant cigarette smoking. Additional oral effects observed with SLT use include extrinsic staining, tooth wear or abrasion, and increased caries prevalence associated with products containing sweeteners or coarse particulates. Regarding malignancy, signals from observational literature indicate an association between SLT exposure and oral squamous cell carcinoma (OSCC), although the magnitude of risk appears to vary by product chemistry (e.g., nitrosamine content, pH), processing methods, and regional usage patterns. Evidence remains heterogeneous, limiting direct comparisons across products and populations. Small biomarker studies also report elevated salivary pro-inflammatory cytokines (IL-6, IL-8, and TNF-α) among users with mucosal changes, but clinical utility is uncertain. Overall, current evidence links SLT with frequent, often reversible local mucosal alterations, consistent placement-site gingival recession, and a possible product-dependent elevation in OSCC risk. These observations underscore the importance of establishing uniform definitions for outcomes and consistent measures of exposure in studies of SLT to facilitate valid comparisons across different products and populations. In addition, further research is warranted to explore the motivations for SLT use and to delineate dose-response relationships and potential malignant transformation, with particular attention to emerging nicotine pouch products.

## Introduction and background

Tobacco products can be divided into two categories: smoking tobacco and smokeless tobacco (SLT). SLT encompasses a wide range of products, including naswar, betel quid, snuff, and gutkha, that are consumed without combustion. Instead of being burned, these products are used by chewing, placing between the lip and the gum (dipping), or inhaling them into the nasal cavity (snuffing) [1]. According to the World Health Organization’s Framework Convention on Tobacco Control, SLT is defined as tobacco consumed in its unburnt form via oral or nasal routes [2].

SLT products are known by various names across the world, with each type undergoing a distinct preparation process. In the US, powdered dry snuff, loose-leaf chewing tobacco, and moist snuff are traditional types of SLT products. In Scandinavian countries, especially in Sweden, snus is commonly used; it is a moist oral tobacco available in loose form or small, portioned pouches. Snus is placed behind the upper lip [3]. Unlike snus, which is usually pasteurized and packaged in pouches, many SLT products used in South Asia are fermented during processing. Furthermore, in Sweden, the production of snus follows national food-grade regulations and adheres to the strict industry standard known as "GothiaTek." Indian SLT products contain betel leaf (Piper betle), sliced areca nut (Areca catechu), and powdered agricultural lime. The presence of these additives, in conjunction with the fermentation process, significantly contributes to the carcinogenicity, toxicity, and psychotropic potential of these SLT products [4].

Globally, SLT is consumed in 127 countries [5], with an estimated 365 million users [1]. The vast majority of these users reside in South and Southeast Asia [5]. The prevalence of SLT use among men varies, reaching 30% in India, 20% in Sweden, and 6% in Iceland [6,7]. In many low- and middle-income countries, SLT consumption is also more prevalent among women compared to cigarette smoking. This trend is induced by acceptability and the common misconception that it is a less harmful alternative to smoking [5]. Moreover, a study by Yanyun He et al. found that despite a low average tax burden on SLT consumers, tobacco companies fully, or even excessively, transfer these costs to the prices of their premium products. Therefore, the authors recommend implementing minimum pricing and restricting promotions to effectively increase the cost of cheaper goods and enhance the effectiveness of the tax policy [8].

SLT products are highly addictive and contain various carcinogens such as nitrosamine acids, arsenic, nickel, cadmium, beryllium, nitrate, nitrite, and chromium [9]. The chemical composition also depends on the manufacturing process. In India, the fermentation of SLT products leads to the production of potential carcinogens called tobacco-specific nitrosamines (TSNAs) [7]. These products often differ in terms of pH, with higher pH values increasing nicotine absorption, thereby enhancing the risk of addiction. Raising the pH is also associated with greater absorption of carcinogens, resulting in increased toxicity [9]. Globally, the use of SLT products is responsible for more than 650,000 deaths annually [2].

Methods

The goal of this review was to offer a thorough synthesis of current scientific evidence regarding the effects of SLT products, including snus, chewing tobacco, naswar, toombak, gutka, betel quid, and nicotine pouches, on oral health. A comprehensive literature search was carried out in PubMed/MEDLINE, Scopus, Web of Science, and the Cochrane Library. In addition, Google Scholar was examined to identify grey literature and studies specific to certain regions that were not indexed in the main databases. The search strategy combined Medical Subject Headings (MeSH) and free-text keywords. 

Exposure-specific terminology featured “Smokeless Tobacco”[MeSH], “Tobacco, Smokeless”[MeSH], “Tobacco Use”[MeSH], together with keywords “snus,” “chewing tobacco,” “naswar,” “toombak,” “gutka,” “betel quid,” and “nicotine pouch.” Outcome-related terms included “Oral Mucosa”[MeSH], “Mouth Diseases”[MeSH], “Leukoplakia, Oral”[MeSH], “Periodontitis”[MeSH], “Carcinoma, Squamous Cell”[MeSH], and free-text keywords such as “oral lesion,” “keratosis,” “gingival recession,” “dental caries,” “oral squamous cell carcinoma,” “oral cancer,” “cytokines,” “IL-6,” “IL-8,” and “TNF-alpha.” Boolean operators (AND/OR) were used to combine search terms appropriately.

Eligibility criteria

Studies were deemed suitable for inclusion if they were observational (e.g., cross-sectional, cohort, or case-control), systematic reviews, meta-analyses, or biomarker studies, and comprised human participants of any age who used SLT. The relevant outcomes consisted of oral health manifestations such as mucosal changes, periodontal disease, dental caries, malignant or premalignant lesions, or biomarker shifts. Inclusion was limited to peer-reviewed articles written in English and published from January 1990 to March 2025.

Studies were excluded if they were animal or in vitro studies, case reports or series with fewer than five participants, or studies solely examining cigarette smoking without providing comparative data on SLT.

Study selection and data extraction

The selection process involved independent checking of titles and abstracts by two reviewers, followed by full-text evaluation for eligibility. Any inconsistencies were resolved through discussion.

We extracted the following data from the included research: author, year, country, study design, sample size, features of the study population, manner and type of SLT use (product, frequency, duration, placement location), oral health endpoints examined, principal findings (e.g., prevalence, odds ratios), and statistical adjustments for confounders including cigarette smoking, alcohol consumption, and oral hygiene regimens.

Data synthesis

The variability in study designs, how exposure was defined, and the chemical composition of products meant that a meta-analysis could not be performed. Instead, results were synthesized narratively and grouped into four thematic domains: mucosal alterations, periodontal effects, malignant transformation, and biomarker and mechanistic evidence. 

## Review

Mucosal changes

Oral mucosal lesions are defined as abnormal changes or swelling affecting the epithelial lining of the mouth, lips, or gums, which do not contain any malignant or pre-malignant cells [3]. Redness, stinging, itching, or a burning sensation in the oral mucosa can be manifested by repeated placement of a nicotine pouch under the upper lip. These changes can extend to the entire oral cavity and throat.

The inability to perceive tastes can also be revealed. Some users of snuses struggle with more disturbing modifications, including ulcers, canker sores, or changes in saliva production.

These alterations are a consequence of impaired blood flow within the oral mucosa, a decreased white blood cell count, and long-term exposure to the triggering agent [10]. A study performed on young adults in Norway in 2015 and 2016 revealed that as many as 79.2% of daily snus users experienced the previously mentioned oral mucosal alterations. Moreover, gum recession was observed in over 18% of the participants, as reported by the study's authors. Connective tissue loss occurring at the site of snus placement is a reason for these changes [10].

The investigation revealed mucosal changes in nearly 12% of snus-using respondents, who showed a tendency to experience these alterations [11]. Respondents affirmed that visible mucosal alterations were observed at the sites of snus or nicotine pouch placement. Some of the respondents had white, round, dot-like lesions, which were leathery and placed from tooth number 13 to 22. One of the respondents exhibited whitish, line-like lesions in the sublabial vestibule. Higher risk of mucosal alterations was reported after five to 10 years of using tobacco products. An association was also observed between mucosal changes and the daily consumption of tobacco units. It can be a tendency to experience mucosal changes in people who use five to 10 tobacco units per day. Referred to as SLT keratosis [12], these white lesions typically appear in their early stages with a white appearance and minimal mucosal thickening. During the progression of the lesion, the mucosa becomes more keratotic and thicker [13].

Fifteen Scandinavian studies have shown a link between SLT and oral mucosal lesions. Findings consistently show that snuff-induced lesions (SILs) are present in nearly all individuals who currently use moist snuff, and their severity is correlated with the intensity and duration of use [14]. The authors of this study [15] compared the responses of Scandinavian and American populations to SLT, referencing 24 US-based studies that documented a consistent association between SLT use and the development of mucosal lesions at sites of product placement. It is unconditionally clear that the usage of snuff notably enhances the predisposition to oral mucosal alterations. Among Scandinavian users, the prevalence of the typical SIL approaches 100%, whereas this lesion is absent in non-users. By contrast, the spectrum of lesions examined in US studies is considerably broader. The presence of mucosal alterations is not exclusive to snuff users, as they may also arise in non-using populations. The frequency of occurrence is significantly higher in users than in non-users, with published odds ratios of up to around 100. While strong dose-response patterns are evident with general SLT use, the correlation between active chewing tobacco use and lesion occurrence, largely investigated in the United States, appears considerably weaker. In both regions, the occurrence of oral mucosal lesions among previous users of snuff, chewing tobacco, or general SLT remains low and does not exceed that seen in individuals without any history of tobacco use. It may suggest the recoverability of the effect. This is consistent with the results of previous research showing a precipitous decline after quitting, as well as an abrupt onset in individuals who began using SLT. As studies indicate, the stage of a disease may influence a change in the SLT product [15].

Leukoplakia 

Another potential consequence of snus use is the development of leukoplakia, a potentially malignant disorder marked by white lesions within the oral mucosa [16]. This oral mucosal alteration is of particular concern as it serves as a warning sign because of its potential to progress into squamous cell carcinoma (SCC) [17,18]. The discrepancies reported in the literature may appear from variations in study methodologies. A study demonstrated that the use of snus was linked to decreased accumulation of dental calculus on anterior teeth, most prominently in regions adjacent to the usual position of the pouch [19]. This finding has been linked to the greater salivation in specific areas, together with recurrent mechanical tongue stimulation, which explains this observation [10].

The diagnosis of leukoplakia is most commonly made in individuals over 40 years of age. Its occurrence is higher in males and roughly sixfold greater among individuals who use SLT than in those who do not consume tobacco. In SLT users, the buccal mucosa and lower buccal grooves are the sites most often affected, primarily due to the habitual placement of the tobacco quid in these locations [20]. Clinically, leukoplakia is classified into two main types determined through surface appearance and morphological properties, such as structural density and appearance: homogeneous versus heterogeneous. Homogeneous leukoplakia presents as a uniformly flat and thin white lesion. However, the non-homogeneous type comprises three clinical types: speckled, nodular, and verrucous. The speckled type is mixed, white and red, so it can also be termed erythroleukoplakia. Nodular variant is a small polypoid outgrowth, a rounded red or white excrescence. The third type, verrucous, has a wrinkled or corrugated surface. In most cases, leukoplakias are asymptomatic. The presence of a red component within erythroleukoplakia may indicate colonization by *Candida *species [21]. Diagnosis of leukoplakia remains provisional until other white lesion-causing conditions are ruled out.

Recent findings summarized by the US Surgeon General [15] describe leukoplakia as a prototypical oral cancer precursor lesion, observing that "the leukoplakia that occurs in cigarette smokers differs morphologically from the keratoses caused by SLT; although less common, the leukoplakia induced by cigarettes is more susceptible to malignant transformation," as highlighted in Bouquot’s review [22]. Several reviewers have likewise underscored the significance of separating SLT-related lesions from classical oral leukoplakia [15,23,24].

Gingival recession

Gum disease is a prevalent condition characterized by swelling, soreness, or infection of the gums. The early stage of gum disease is known as gingivitis. The condition may worsen into periodontal disease, a serious disorder affecting both the jawbone and the tissues that support the teeth. There are eight relevant Swedish publications that examined the relationship between snus exposure and diverse clinical manifestations of periodontal disease [3]. Findings from one study indicated that snus users exhibited a significantly higher gingival index, a measure of gum inflammation [25]. By contrast, several other studies did not find any association between snus use and gum infection, inflammation, or bleeding. Five of the seven publications considered the issue of gum recession [26-28]. One study reported a significant increase in receding gums among adolescent snus users compared with controls, while another study observed that 23.5% of individuals who have used loose snus experienced receding gums, compared to only 2.9% of those who have used snus pouches [25]. However, three publications reported that the use of snus did not significantly correlate with gum recession [26-28]. The most extensive publication, based on three epidemiological cross-sectional studies, investigated the potential negative effects of smoking and snus use on periodontal health compared to individuals not using tobacco. According to the study, smoking cigarettes exposes users to a greater risk of advanced periodontitis in contrast to the group using non-tobacco products and snus. The study concluded that there was no statistically significant association between snus usage and the development of periodontitis [29].

Gum bleeding is also an alarming symptom of using SLT, and it is a primary symptom of gingivitis [30,31]. One of the studies underscored the cumulative impact of inadequate oral hygiene and regular snus use, which is implicated in increased gum bleeding. Inflammation, edema, redness, and bleeding are the characteristics of gingivitis, which is caused by the accumulation of bacterial biofilm [32,33]. SLT is associated with a higher incidence of caries and abrasion, which causes discoloration of teeth, and regular consumers tend to have more tooth loss [34].

According to the study, users of SLT demonstrate poorer periodontal conditions compared to non-users. The detrimental effects of SLT on periodontal tissues are primarily attributed to the harmful substances contained within it [35]. Arecanut, as one of its components, contains an alkaloid known as arecoline, which reduces the proliferation of gingival keratinocytes and fibroblasts, thereby reducing collagen synthesis [36]. The likelihood of gingival recession among SLT users was seen to be 1.71 times higher. This difference in the strength of association, relative to pocket depth, may be explained by the fact that pocket formation typically precedes gingival recession in the progression of periodontal disease. However, one of the well-known side effects of these products is localized gingival recession near where the product is held, and we might anticipate that we will see similar effects in nicotine pouch users due to their similar methods of administration [37]. The use of chewing tobacco and SLT products causes considerable staining of dental surfaces. Tooth discoloration is frequently caused by chewing tobacco. Brown-to-black staining develops as deposits adhere to and infiltrate dental tissues. Long-term chewing tobacco users may also be more exposed to the discoloration of prosthetic dentition and prosthetic devices. Dental erosion and abrasion are outcomes of chewing SLT products, which contain coarse abrasives. Moreover, chewing tobacco users are more likely to develop tooth decay, which can lead to tooth loss. Epidemiological data suggest that tobacco users experience a 67% higher rate of tooth loss compared to non-users. The high content of sweetening and flavoring agents in tobacco products is responsible for dental decay [38].

With regard to the gingiva and periodontal tissues, the use of SLT is associated with an increased prevalence of gingival recession with exposure of the tooth root surface, periodontal pocket formation, the accumulation of plaque and calculus, which leads to periodontitis [16]. The gingival recession tends to be more prominent in the anterior region of teeth [39].

Various reviews suggest a possible influence on gingival recession, and a controversy exists regarding the clear demonstration of causal link [40-42]. The conclusions regarding a relationship with periodontal diseases and dental caries are mixed, with some studies suggesting no association while others indicate a clear link [43]. Multiple reviews have noted the reversibility of oral mucosal lesions following the cessation of SLT use; they then address the role of these lesions as a modifier of oral cancer risk [15].

SLT and oral cancer

Oral Cavity Cancer

Oral cavity cancer (OCC) is a subclass of head and neck cancer and globally is the sixth most common cancer [44,45]. It is located in the mouth and the back of the throat. The most common areas where cancer appears are the tongue, under and at the base of the tongue, on the tissue lining the mouth and gums, and on the throat at the back of the mouth [46]. In the year 2020, oral cancer was responsible for 177,757 deaths. The five-year survival rates for OCC vary significantly, ranging from 39% to 84% depending on the stage of the disease and between 48% and 67% across ethnic groups [44].

People over the age of 40 are being exposed to oral cancer. Other risk factors are the sex of male, usage of SLT products, drinking alcohol, and human papilloma virus (HPV) [46].

SLT products contain a large amount of toxic ingredients and carcinogens; the number of them is strongly correlated with the manufacturing process, such as fermentation of SLT products, which leads to the formation of tobacco-specific nitrosamines (TSNAs) [7]. The presence of carcinogens leads to mutation in K-ras, p53, and other genes caused by the formation of the DNA adducts [47]. The metabolic activation of carcinogens may also indicate chronic local inflammation, oxidative stress, and tumor promotion. These mechanisms and others, such as reduced apoptosis or cellular transformation, may also increase the occurrence of cancer. Another cancer risks are epigenetic pathways causing unregulated proliferation [48].

Based on the systematic review by Gupta et al., the cohort evidence is mixed [49]. Of five cohort studies, three reported a positive association between SLT use and the risk of oral cancer. Positive findings were observed mainly in studies from Southeast Asia, whereas European cohorts generally did not detect an increased risk. Two studies specifically linked tobacco chewing with a higher risk of OCC. Evidence for snus was inconsistent: in two of four studies, no positive association with OCC was found. Most case-control studies reported a significant positive association between SLT use and oral cancer, while some did not support this link [49]. Several studies provided sex-specific estimates and found significantly elevated risks in both men and women; some suggested a stronger effect in women (OR ~3.2-45.89) than in men within the same studies (OR ~2.7-9.33) Regarding product types, most estimates concerned chewing products (e.g., gutka, betel quid/paan with tobacco, zarda, khaini, and mishri). In most of these, a positive association was observed, although at least one study did not find such a link. Where exposure was described only as “tobacco chewing” without specifying the product, results were mixed. Analyses involving toombak and naswar reported significant positive associations. For snuff, findings were inconsistent: some studies indicated a higher risk, whereas others did not. Notably, a subset of studies did not adjust for cigarette smoking as a potential confounder, which should be considered when interpreting these results.

Oral Squamous Cell Carcinoma

The primary type of oral cancer, accounting for over 90% of cases, is SCC, which develops from non-healing ulcerations and premalignant conditions (lichen planus and leukoplakia) [50,51]. Its development is promoted by viral infections (EBV and HPV), poor oral hygiene, or genetic and dietary factors [52,53]. However, for users of SLT, the risk increases by more than 16-fold [54]. Geographically, oral squamous cell carcinoma (OSCC) occurs much more frequently in the countries of South Asia and the Pacific region than in Europe or North America [55,56]. This aggressive cancer, affecting the oral cavity, lips, and oropharynx, is one of the most common worldwide and is classified as a head and neck cancer [57]. Clinically, OSCC is most often located in the regions of the floor of the mouth, lips, and tongue [58]. Unfortunately, many early-stage lesions are often overlooked due to the painless, paucisymptomatic, and insidious nature of this cancer. Consequently, nearly half of all OSCC cases are diagnosed only in an advanced phase (stage III or IV). This translates into a significantly worse prognosis for the patient and is the cause of a high mortality rate [59-61].

The use of SLT is a recognized risk factor in the context of these cancers, which can lead to the development of specific premalignant lesions. A cohort study by Critchley et al. [62] observed the development of white lesions, also known as SLT keratosis [12], in the oral mucosa of snus users. They occurred primarily in men using a minimum of five to 10 sachets per day for five to 10 consecutive years. The study showed that the absolute risk of malignant transformation of these lesions was low, and that in most cases, they resolve completely after cessation of product use. However, this risk is not zero. Therefore, any lesions in the oral mucosa that do not resolve over a long period or that exhibit features of induration and ulceration should undergo histopathological evaluation to rule out the development of SCC.

SLTs have an aggressive effect on the oral mucosa, resulting in its surface becoming diffuse, wrinkled, thickened, and corrugated. Over time, these changes can develop into SCC [63]. The use of SLT increases the risk of developing OSCC, particularly among women, representing a significant social and public health concern [44].

Quantities of pro-inflammatory cytokines such as interleukin-1 (IL-1), IL-6, IL-8, TNF−α, and leucine rich alfa-2 glycoprotein (LRG-1) are higher in the saliva of individuals using various forms of tobacco [11]. In individuals diagnosed with mucosal changes, an elevated level of IL−6 in saliva was also detected. Conversely, the analysis showed that the LRG-1 marker is not useful as a prognostic indicator in this context [11]. In the Miluna et al. study [11], the highest values of IL-1, IL-6, IL-8, TNF alpha, and LRG1 levels were found in the snus group; these are also close to mucosal changes in this group, and the snus group exhibited the peak LRG-1 protein content. Research by others, focused on quantifying salivary IL-6, IL-8, and TNF-alpha in patients with oral leukoplakia, submucous fibrosis, and lichen planus (all confirmed by histology), revealed a significant escalation in these biomarker concentrations [64]. The research by Kawahara et al. indicated a correlation between leucine rich alfa-2 glycoprotein concentrations and the propensity for OSCC [65]. Moreover, in the study by Miluna et al., an association was found between elevated IL-6 levels linked to tobacco consumption. Salivary IL-6 subsequently triggers the activation of Janus kinases (JAK) and signal transducers and activators of transcription (STATs). They initiate a route that activates mitogen-activated protein kinase (MAPK); this pathway promotes cell multiplication and angiogenesis and plays a role in the advancement of cancer [11]. Regarding cellular processes, IL-8 exerts an effect on cell growth and neovascularization, while TNF-alpha governs cell survival, programmed cell death, and the multiplication of cells, and all of these processes are involved in oral cancer development [66].

SLT and Periodontitis

Another potential consequence of SLT use is periodontitis. It is an infectious-inflammatory disease that develops through an inflammatory process, with biofilm formation as a key factor. The inflammatory response affects the gingiva and leads to loss of clinical attachment of periodontal tissues and alveolar bone [67]. Globally, periodontitis ranks as the 11th most common health problem and contributes to 3.5 million years lived with disability [68]. In its severe stage, it constitutes the sixth leading cause of tooth loss worldwide, and its progression impacts not only physiological but also psychological and social aspects of oral health, thereby adversely affecting patients’ quality of life [35,69]. Initiation and progression of periodontitis are associated with age, sex, low socioeconomic status, poor oral hygiene, uncontrolled diabetes, and tobacco use [68].

Data from 19 studies indicate that SLT users have a significantly higher prevalence of periodontitis than non-users [68]. The risk is elevated nearly threefold (OR ≈ 2.99), with no association between the specific type of SLT product and the risk. Periodontal pocket depth (PPD) was assessed in 21 studies conducted in Asian populations, and three additional studies evaluated radiographic marginal bone loss. Compared with non-users, SLT users showed >3.5-fold higher odds of PPD >4 mm in 18 studies [68]. Ten studies reported nearly 3.5-fold higher odds of PPD >6 mm among SLT users [68]. Six studies from Asian countries reported gingival recession among SLT users, showing approximately 1.7-fold higher odds compared with non-users [68]. Five studies assessed clinical attachment level (CAL) in SLT users, and another five focused on loss of attachment (LOA) [68]. All reported >2.8-fold higher odds of LOA of 4-5 mm. LOA ≥6 mm was described in three of the five studies, with SLT users exhibiting roughly fourfold higher odds than non-users [68].

## Conclusions

SLT - including snus, chewing tobacco, naswar, and nicotine pouches - produces consistent, predominantly local effects in the oral cavity. The most characteristic changes are placement-site mucosal lesions (smokeless-tobacco keratosis and white plaque-like lesions) and localized gingival recession; many of these alterations improve or resolve after discontinuation. Evidence for broader periodontal destruction is mixed and frequently confounded by oral hygiene and concurrent cigarette smoking. Additional consequences - extrinsic staining, tooth wear/abrasion, and higher caries experience where products contain sweeteners or coarse particulates - reflect both product chemistry and user behavior. With respect to malignancy, the balance of observational data supports an elevated, product- and region-dependent risk of OSCC, strongest in settings that use fermented, high-nitrosamine mixtures; nevertheless, heterogeneity in exposure and outcome definitions limits precise risk quantification.

The clinical implications of these findings are unequivocal. Health professionals should routinely inquire about SLT, inspect habitual placement sites, document and photograph lesions, screen for periodontal disease (PPD/CAL/LOA), and provide brief advice and structured cessation support. Any persistent, indurated, ulcerated, or non-homogeneous (erythroleukoplakic or verrucous) lesion warrants a timely biopsy. Patients should be counselled that “smoke-free” does not mean “risk-free” and that regression of keratotic lesions after quitting does not preclude ongoing surveillance. Interdisciplinary pathways between dentistry, primary care, and head-and-neck oncology can shorten time to diagnosis for suspicious lesions and improve follow-up of high-risk users. While salivary inflammatory markers are of research interest, they should not replace clinical examination and histopathology.

Future work should prioritize standardized exposure metrics (product type, pH, nitrosamine content, placement habits), harmonized periodontal outcome definitions (PPD thresholds, CAL, and LOA), and adequately powered longitudinal studies to clarify dose-response and malignant transformation rates. Comparative evaluation of newer nicotine pouches versus traditional SLT products is needed, given rapid uptake and uncertain long-term effects. Public health measures - accurate risk communication, warning labels, age restrictions, and product standards that limit carcinogen content - can complement clinical efforts. Together, these steps offer a pragmatic pathway to reduce SLT-related oral morbidity and to detect OSCC earlier in populations where use remains common.
